# Diagnosis and Treatment Effect of Convolutional Neural Network-Based Magnetic Resonance Image Features on Severe Stroke and Mental State

**DOI:** 10.1155/2021/8947789

**Published:** 2021-07-26

**Authors:** Lihong Han, Li Liu, Yankun Hao, Lan Zhang

**Affiliations:** ^1^Department of Medical Education, The First Affiliated Hospital of Jiamusi University, Jiamusi 154002, Heilongjiang, China; ^2^Department of Emergency, The First Affiliated Hospital of Jiamusi University, Jiamusi 154002, Heilongjiang, China; ^3^Department of Medical Function, Mudanjiang Medical University, Mudanjiang 157011, Heilongjiang, China; ^4^Department of Student Affairs, Mudanjiang Medical University, Mudanjiang 157011, Heilongjiang, China

## Abstract

The purpose of this paper is to explore the impact of magnetic resonance imaging (MRI) image features based on convolutional neural network (CNN) algorithm and conditional random field on the diagnosis and mental state of patients with severe stroke. 208 patients with severe stroke who all received MRI examination were recruited as the research objects. According to cerebral small vascular disease (CSVD) score, the patients were divided into CSVD 0∼4 groups. The patients who completed the three-month follow-up were classified into cognitive impairment group (124 cases) and the noncognitive impairment group (84 cases) according to the cut-off point of the Montreal cognitive assessment (MOCA) scale score of 26. A novel image segmentation algorithm was proposed based on U-shaped fully CNN (U-Net) and conditional random field, which was compared with the fully CNN (FCN) algorithm and U-Net algorithm, and was applied to the MRI segmentation training of patients with severe stroke. It was found that the average symmetric surface distance (ASSD) (3.13 ± 1.35), Hoffman distance (HD) (28.71 ± 9.05), Dice coefficient (0.78 ± 1.35), accuracy (0.74 ± 0.11), and sensitivity (0.85 ± 0.13) of the proposed algorithm were superior to those of FCN algorithm and U-Net algorithm. There were significant differences in the MOCA scores among the five groups of patients from CSVD 0 to CSVD 4 in the three time periods (0, 1, and 3 months) (*P* < 0.05). Differences in cerebral microhemorrhage (CMB), perivascular space (PVS), and number of cavities, Fazekas, and total CSVD scores between the two groups were significant (*P* < 0.05). Multivariate regression found that the number of PVS, white matter hyperintensity (WMH) Fazekas, and total CSVD score were independent factors of cognitive impairment. In short, MRI images based on deep learning image segmentation algorithm had good application value for clinical diagnosis and treatment of stroke and can effectively improve the detection effect of brain domain characteristics and psychological state of patients after stroke.

## 1. Introduction

Severe stroke is a cerebrovascular disease with high morbidity, disability, and mortality. Studies showed that the prevalence of cognitive impairment in patients with severe stroke was abnormally as high as 37% to 80%, and severe cases can be fatal [1, 2]. Therefore, the early diagnosis of severe stroke is particularly important. Generally, doctors perform magnetic resonance imaging (MRI) to diagnose severe stroke lesions, but it takes a long time to artificially segment severe stroke lesions and this process consumes manpower, material, and financial resources. The most severe problem is that it is affected by the subjective consciousness of medical staff [3]. Therefore, it is very necessary to propose a high-efficiency and high-accuracy image segmentation algorithm. So far, convolutional neural network (CNN) algorithms have been widely used in medical image processing and lesion segmentation and have achieved remarkable results. Not only can they automatically extract the features in the data but also they have objectivity and efficiency compared with machine learning algorithms. Puri and Cox [4] proposed U-Net, which combines low-level information with high-level information to obtain an ideal segmentation effect. To make better use of the deep information of the three-dimensional image, the CNN can be adopted to propose an algorithm that can quickly and accurately segment the MRI images of patients with severe stroke, which will be helpful for medical workers to diagnose and treat patients with severe stroke early.

Cerebral small vascular disease (CSVD) is a common cerebrovascular disease that plagues the elderly. It can cause lacunar infarction, cerebral hemorrhage, and so forth, accounting for 20% of symptomatic strokes. 35% of Alzheimer's disease is closely related to CSVD [5, 6]. Studies showed that patients with severe stroke often have different degrees of imaging features, which can lead to cognitive impairment and affect their later living conditions [7, 8]. To reflect its impact on the brain, Nylander et al. [9] proposed the concept of “total CSVD score.” Many studies revealed that the total CSVD score can well reflect the overall impact of imaging features on the brain and was closely related to the cognitive impairment of stroke patients. However, there are few reports on the characteristics of cerebrovascular disease in patients with CSVD and the impact of CSVD score on cognitive impairment after severe stroke. Therefore, a new image segmentation algorithm was proposed based on U-Net algorithm and conditional random field algorithm, which was compared with FCN algorithm and U-Net algorithm. Then, it was applied to MRI diagnosis of 208 patients with severe stroke in our hospital to study the relationship between MRI image features and CSVD score and cognitive impairment after severe stroke. It aimed to decrease the occurrence of cognitive impairment in severe stroke patients and perform early detection and early diagnosis, thereby alleviating the cognitive impairment in severe stroke patients.

## 2. Materials and Methods

### 2.1. Research Objects

A total of 208 severe stroke patients who were admitted to the hospital from January 2017 to October 2020 were selected, 129 males and 79 females, aged 40–80 years. According to the total CSVD score, patients were rolled into CSVD 0–4 groups. Montreal cognitive assessment (MOCA) scale score of 26 was the cut-off point. The patients who received the 3-month follow-up were rolled into cognitive impairment group (124 cases) and noncognitive impairment group (84 cases). The study had been approved by the Medical Ethics Committee of the Hospital, and an informed consent form had been signed.

Inclusion criteria were as follows: (i) the definition of severe stroke being 21 points ≤ National Institutes of Health Stroke Scale (NIHSS) scores ≤ 42 points according to the diagnostic criteria and confirmed by imaging examination as cerebral infarction [10]; (ii) age ≥18 years; (iii) those who can cooperate with medical staff to perform scale scoring and MRI examination; (iv) the basic information being complete, and the most important thing was that the patient was willing to cooperate with the follow-up; and (v) patients who signed the informed consent.

Exclusion criteria were as follows: (i) patients with central nervous system disease; (ii) patients with history of drug abuse and alcohol abuse; (iii) patients who had a family history of dementia; (iv) patients with poor compliance; (v) patients who withdrew during the experiments; (vi) patients who cannot undergo imaging examination due to surgical implantation of organs; (vii) recurrence and death that occurred during the follow-up; and (viii) patients with anxiety and depression [11].

### 2.2. Imaging Examination

Signa HD 3.0T high-field superconducting magnetic resonance scanner from GE Company was employed to perform head MRI and MRA inspections. The brain MRI imaging sequence included (i) T1-weighted spin echo axial image (T1WI): time repetition (TR)/time echo (TE) was 1750/24 ms; (ii) T2-weighted spin echo axial image (T2WI): TR/TE was 3180/110 ms; (iii) liquid attenuation reversal recovery sequence (FLAIR): TR/TE was 8000/140 ms; (iv) diffusion weighted imaging (DWI): TR/TE was 5300/81.4 ms; and (v) susceptibility weighted imaging (SWI): *b* = (0, 1000). The scanning parameters were as follows: layer thickness of 5 mm, layer spacing of 1.5 mm, field of view (FOV) of 240 × 240 mm, and matrix of 310 × 246. The coronal and horizontal axis positions of TIWI and T2WI and the horizontal axis positions of T2-FLAIR, DWI, and SWI were collected. Two experienced radiologists and neurologists were selected for review.

MRI features included white matter hyperintensity (WMH) that may be vascular origin, cerebral microhemorrhage (CMB), perivascular space (PVS), and lacuna that may be of vascular origin. The image features and scoring criteria are shown in Table 1.

### 2.3. Image Segmentation Algorithm Based on U-Net and Conditional Random Field

CNN developed based on neural networks has very superior performance in medical image processing. A new image segmentation algorithm was proposed in this research based on the U-Net and fully connected conditional random field. The U-Net network architecture consists of an encoding path and a decoding path. The encoding path can obtain spatial information, which is composed of multiple convolutional layers and downsampling layers. The decoding path can restore the input resolution and performs subconvolution and upsampling of these features. The convolution process is as follows:

(1) $$\begin{array}{c} a_{j}^{n}=f\left(\sum_{i\in M_{j}}a_{j}^{n-1}\cdot k_{ij}^{n}+b_{j}^{n}\right). \end{array}$$

In equation (1), *k* is the convolution kernel, *n* is the number of layers, *k*_*ij*_^*n*^ is the weight, *b* is the bias value, and *a*_*j*_^*n*−1^ is the output of the previous layer. The sampling expression in the pooling layer is as follows:

(2) $$\begin{array}{c} a_{j}^{n}=f\left[\beta_{j}^{n}\text{down}\left(a_{j}^{n-1}\right)+b_{j}^{n}\right]. \end{array}$$

In equation (2), down(·) is the downsampling function, while *β* and *b* denote the multiplicative bias and additive bias, respectively.

The algorithm proposed has an extra clipping layer and a multiloss structure, which can segment images of any size and solve the disappearance of gradients. In addition, the 1 × 1 convolution layer and softmax function of the convolution kernel are added, whose weights are 0.8 and 0.2, respectively. The fully connected layer is expressed as follows:

(3) $$\begin{array}{c} a^{n}=f\left(w^{n}a^{n-1}+b^{n}\right). \end{array}$$

In equation (3), *w*^*n*^ is the weight coefficient, *a*^*n*−1^ is the output of the previous layer, and *b*^*n*^ is the bias term. Softmax can finish more than two types of classification tasks. The calculation equation is as follows:

(4) $$\begin{array}{c} h_{\left(\omega \right)}\left(c^{i}\right)=\left[\begin{array}{c} P\left(d^{i}\right)=1\left|c^{i},\omega \right)\\ P\left(d^{i}\right)=2\left|c^{i},\omega \right)\\ \ldots \\ P\left(d^{i}\right)=k\left|c^{i},\omega \right) \end{array}\right]=\frac{1}{\sum_{j=1}^{k}e^{\omega_{k}^{T}c^{i}}}\left[\begin{array}{c} e^{\omega_{1}^{T}c^{i}}\\ e^{\omega_{2}^{T}c^{i}}\\ \ldots \\ e^{\omega_{k}^{T}c^{i}} \end{array}\right]. \end{array}$$

In equation (4), *c*^*i*^ ∈ *R* is the input sample, *d*^*i*^ ∈ {1,2,…, *k*} is the sample label, *h*_*ω*_(·) is the hypothesis function, *P*(*d*=*j*|*c*) is the probability value, and *ω*=(*ω*_1_*ω*_2_,…, *ω*_*k*_) is the training parameter. After ∑_*j*=1_^*k*^*e*^*ω*_*k*_^*T*^*c*^*i*^^ normalization, the total probability value is 1, and the loss function value of softmax is minimized through training sample *T*. The expression is as follows:

(5) $$\begin{array}{c} J\left(\omega \right)=-\frac{1}{m}\left[\sum_{i=1}^{m}\sum_{j=1}^{k}1\left\{d^{i}=j\right\}\log\frac{e^{\omega_{j}^{T}c^{i}}}{\sum_{i=1}^{k}e^{\omega_{1}^{T}c^{i}}}\right]. \end{array}$$

The binary potential in fully connected CRF is used to improve the accuracy of segmentation. In this algorithm, the Gibbs distribution is expressed as follows:

(6) $$\begin{array}{c} p\left(Y=c\left|I\right.\right)=\frac{e^{-E\left(c\left|I\right.\right)}}{Z\left(I\right)}. \end{array}$$

In equation (6), *I* is the final output of U-Net, *Y*={*Y*_1_,…, *Y*_*N*_} is the category label, and −*E*(*c*|*I*) is the energy function.

(7) $$\begin{array}{c} -E\left(c\left|I\right.\right)=\sum \omega_{i}\left(c_{i}\right)+\sum \omega_{ij}\left(c_{i},c_{j}\right). \end{array}$$

The binary potential energy function is defined as follows:

(8) $$\begin{array}{c} \omega_{ij}\left(c_{i},c_{j}\right)=\mu \left(c_{i},c_{j}\right)\left(\omega_{1}e^{\left(-{\left|p_{i}-p_{j}\right|}^{2}/2\sigma_{\alpha }^{2}\right)-\left({\left|I_{i}-I_{j}\right|}^{2}/2\sigma_{\beta }^{2}\right)}+\omega_{2}e^{\left(-{\left|p_{i}-p_{j}\right|}^{2}/2\sigma_{\gamma }^{2}\right)}\right). \end{array}$$

In equation (8), *p*_*i*_ is the coordinate position of *i*, *I*_*i*_ is the intensity of *i*, |*I*_*i*_ − *I*_*j*_| is the gray value difference, *ω*_1_ and *ω*_2_ are the model weights, and *σ*_*α*_, *σ*_*β*_, and *σ*_*γ*_ are the standard deviation parameters.

Then, the segmentation results are postprocessed as follows: pre(*a*, *b*, *c*) is the voxel (health area: pre(*a*, *b*, *c*)=0, stroke area: pre(*a*, *b*, *c*)=1), and mean_TTP_(*n*) is the average gray value. First, the *n*-th three-dimensional connected domain of mean_TTP_(*n*) > *ω*_1_ is removed, where pre(*a*, *b*, *c*)=0 and *ω*_1_=100. Second, whether it is a lesion area is determined according to the volume of the coarsely segmented lesion. *v*(*n*) represents the volume of the *n*-th connected domain in pre, and *V*_max_ is the volume of the largest connected domain. If *v*(*n*)/*V*_max_=*ω*_2_, remove it, where pre(*a*, *b*, *c*)=0 and *ω*_1_=0.1.

**Figure 1:**
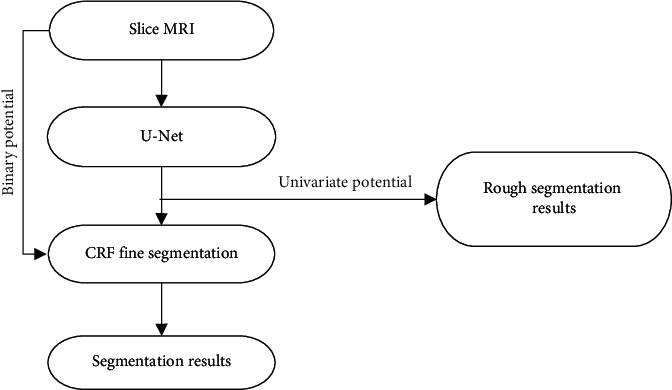
Algorithm flow chart.

From this, a new image segmentation algorithm is constructed, and the algorithm flow is shown in Figure 1.

### 2.4. Network Parameter Configuration

The gradient descent algorithm was adopted for training. In this experiment, the ISLES2015 platform development environment was Matlab2016aCaffe open-source library + cuda8.0 + cuDNN5.1. The operating systems were Windows 10 and Ubuntu 16.04, and the adaptive optimizer Adam [13] was selected. The parameter settings were as follows: the learning rate was 0.0001, the momentum was 0.9, the regularization coefficient was 0.0005, the maximum number of iterations was 21,000, and all biases were initialized to 0.

The collected MRI images of 208 patients were set as training samples, and the algorithm proposed was employed to process the samples. Then, the processing results were uploaded to the above platform for evaluation and were compared with those obtained based on FCN algorithm and the U-Net algorithm. Evaluation indicators included Hoffman distance (HD), average symmetric surface distance (ASSD), sensitivity (recall), Dice coefficient, and precision (precision).

(9) $$\begin{array}{c} \text{HD}\left(E,F\right)=\max\left\{\underset{e\in E}{\max}\underset{f\in F}{\min}D\left(e,f\right),\underset{f\in F}{\max}\underset{e\in E}{\min}D\left(e,f\right)\right\}, \end{array}$$

(10) $$\begin{array}{c} \text{ASSD}\left(E,F\right)=\frac{\text{ASD}\left(E,F\right)+\text{ASD}\left(F,E\right)}{2}. \end{array}$$

In equations (9) and (10), the points on the lesion label and segmentation result are *E* and *F*, respectively, and ASD is average surface distance. Euclidean distance of points *E* and *F* is *D*(*e*, *f*).

(11) $$\begin{array}{c} \text{recall}=\frac{\text{TP}}{\text{TP}+\text{FN}}, \end{array}$$

(12) $$\begin{array}{c} \text{dice}=\frac{2\text{TP}}{\text{TP}+\text{FP}+\text{FN}}, \end{array}$$

(13) $$\begin{array}{c} \text{precision}=\frac{\text{TP}}{\text{TP}+\text{FP}}. \end{array}$$

In equations (11)–(13), TP indicates true positive, FP indicates false positive, and FN indicates false negative.

### 2.5. Neuropsychological Evaluation after Severe Stroke

In this study, the Montreal cognitive assessment (MOCA) scale was used to evaluate the cognitive level of psychology. It is a high-sensitivity and rapid screening scale for cognitive impairment developed by Schellhorn et al. [14] in 2005 with reference to the setting and scoring standards of mini-mental state evaluation (MMSE) cognitive items, and it is based on clinical experience. The test cognitive domains included attention, visual space and executive function, language, naming, delayed recall, abstraction, and orientation, with a total score of 30. A score of 26 was set as a cut-off point. A score lower than 26 was considered to have cognitive impairment, and a score higher than 26 indicated no cognitive impairment. In the subsequent follow-up, if the MOCA score increased by more than two points, it was considered as a certain degree of improvement in cognitive function. Hamilton anxiety rating scale (HAM-A) scoring and Hamilton depression rating scale (HAM-D) scoring were performed to rule out pseudocognitive disorders caused by anxiety and depression [11]. Follow-up was carried out after one month and three months after the stroke. The patient needed to cooperate with two professional neurologists for evaluation.

### 2.6. Statistical Methods

The data obtained was analyzed by SPSS 25.0, and the measurement data were expressed as mean ± standard deviation ( ¯*x* ± *s*). Single-factor logistic regression analysis, multifactor logistic regression analysis, regression analysis coefficient (*R*^2^), and linear regression analysis were utilized.

## 3. Results

### 3.1. Comparison of Training Results of Three Algorithms

**Figure 2:**
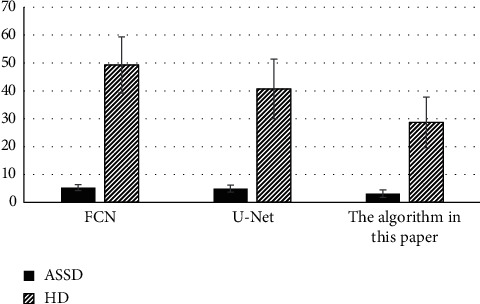
Comparison of ASSD and HD of the three algorithms.

Figure 2 shows the comparison of ASSD and HD among the three algorithms. The ASSD obtained by the algorithm proposed in this research was (3.13 ± 1.35) and the HD was (28.71 ± 9.05), which were inferior to the results obtained by the FCN algorithm and the U-Net algorithm. This meant that the algorithm proposed can automatically segment the stroke lesions with the smallest segmentation error and the highest segmentation accuracy.

**Figure 3:**
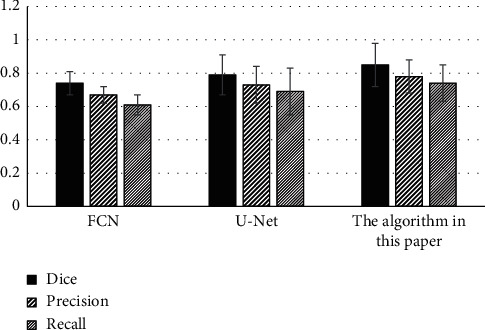
Comparison of Dice coefficient, accuracy, and sensitivity of the three algorithms.

Figure 3 shows the comparison of the Dice coefficient, accuracy, and sensitivity of the three algorithms. The Dice coefficient (0.78 ± 0.1), accuracy (0.74 ± 0.11), and sensitivity (0.85 ± 0.13) obtained by the proposed algorithm were higher than those of the other two algorithms, indicating that the proposed algorithm can automatically segment stroke lesions with the highest segmentation accuracy.

### 3.2. Comparison of MRI Image Segmentation Effects of Three Algorithms

**Figure 4:**
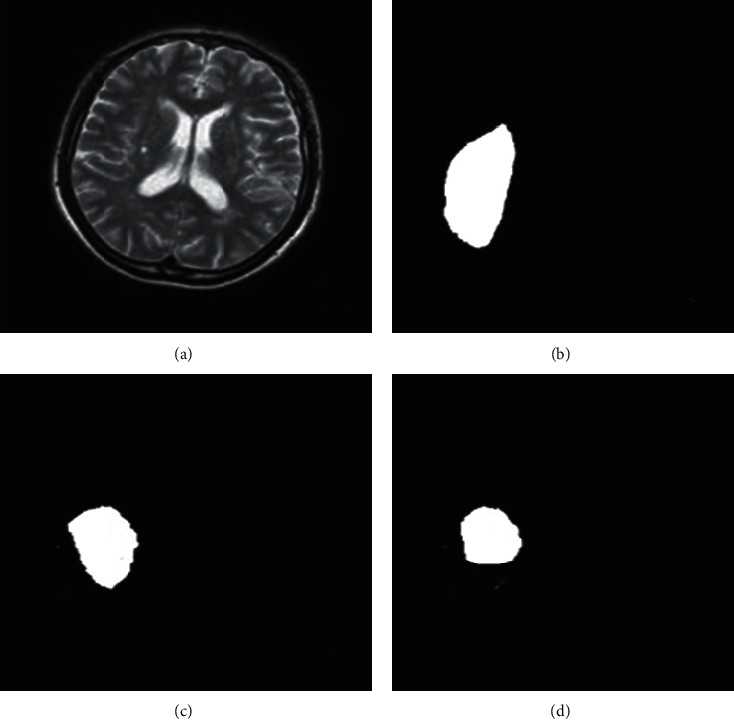
Comparison of the segmentation effects of the three algorithms. (a) Magnetic resonance images of patients with severe stroke were input; (b) FCN algorithm segmentation effect; (c) segmentation effect of U-Net algorithm; (d) segmentation effect of the proposed algorithm.

Figure 4 shows the comparison of the image segmentation effect among the algorithm based on U-Net and conditional random field, the FCN algorithm, and the U-Net algorithm. The proposed algorithm was compared with the FCN algorithm and the U-Net algorithm and applied to the MRI images of 208 severe stroke patients. According to the general structure, the segmentation effect of FCN algorithm and U-Net algorithm was not particularly outstanding, while the segmentation result based on U-Net and conditional random field algorithms was relatively accurate and fine.

### 3.3. Patient's MRI Image Feature Score and Psychological Score

The distribution of MRI features showed that there were 107 cases of WMH (51.4%), 106 cases of CMB (51.2%), 86 cases of lacuna (41.3%), and 87 cases of PVS (41.8%). The distribution of CSVD scores showed that there were CSVD 0 in 25 cases (12%), CSVD 1 in 54 cases (26%), CSVD 2 in 60 cases (28.8%), CSVD 3 in 52 cases (25%), and CSVD 4 in 17 cases (8.2%).

**Figure 5:**
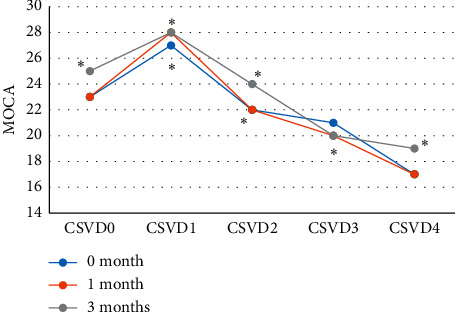
MOCA scores of patients in each group. ^*∗*^ indicates statistical significance (*P* < 0.05).

Figure 5 shows the MOCA scores of patients in each group. MOCA scores of the five groups of patients from CSVD 0 to CSVD 4 were significantly different in three time periods (0, 1, and 3 months) (*P* < 0.05). Then, the correlation between the MOCA score and the total CSVD score at these three times was analyzed. The results suggested that the MOCA scores of 0 months (*r*_*s*_ = −0.675, *P*=0.000), 1 month (*r*_*s*_ = −0.715, *P*=0.000), and 3 months (*r*_*s*_ = −0.655, *P*=0.000) were all negatively correlated with the total CSVD scores, all with significance (*P* < 0.05).

**Figure 6:**
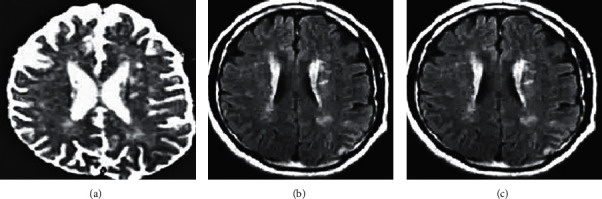
MRI image of a male stroke patient. (a) The diffusion limitation of the left frontal parietal cortex; (b) the diffusion limitation of the subcortical white matter; (c) the edema.

Figure 6 shows the MRI image of a 65 year-old male patient. Epilepsy occurred, associated with altered states of consciousness, acute aphasia, and right staring. Its distribution characteristics did not conform to vascular distribution, edema and gyri enhancement appeared earlier, and cerebral perfusion was normal or increased. There was no vascular occlusion, and sometimes there was both limited cortical diffusion and increased subcortical diffusion.

### 3.4. Explanatory Weight of MRI Feature Score in Cognitive Decline after Severe Stroke

**Figure 7:**
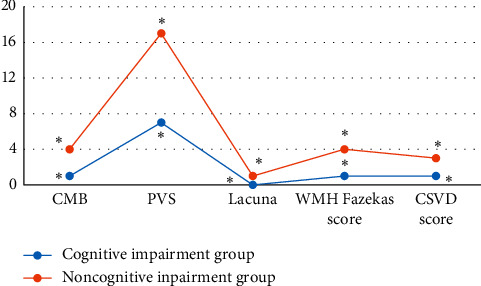
Comparison of the number of CMB, PVS, and lacuna between the cognitive impairment group and the noncognitive impairment group. ^*∗*^ indicates statistical significance (*P* < 0.05).

The patients three months after stroke were rolled into cognitive impairment group (59.6%, 124 cases) and noncognitive impairment group (40.4%, 84 cases) for comparison.  Figure 7 shows that the differences in the number of CMB, PVS, and lacuna, Fazekas score, and total CSVD scores between the two groups were statistically substantial (*P* < 0.05).

**Figure 8:**
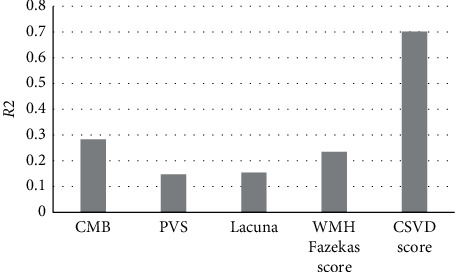
The proportion of CSVD scores affecting cognitive changes.

Linear regression analysis was performed on the variables in Figure 8. Figure 8 shows the explanatory proportions of the number of CMB, PVS, and lacunae, Fazekas score, and total CSVD score to cognitive decline after severe stroke, all of which were significantly correlated with it. Among them, the total CSVD score had the highest explanatory degree (0.702), while the influence of other factors on cognitive decline was generally low.

### 3.5. Correlation Analysis between MRI Image Feature Score and Cognitive Impairment Group

**Figure 9:**
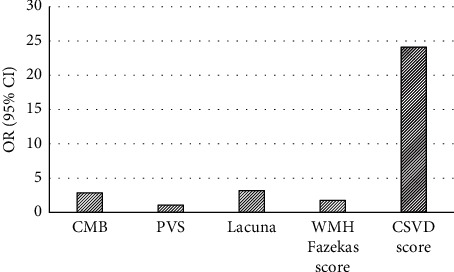
Single-factor binary logistic regression analysis.

Figure 9 shows the single-factor binary logistic regression analysis of the *P* < 0.05 variables of cognitive impairment. With the number of CMB, the number of PVS, the number of cavities, the WMH Fazekas score, and the total CSVD score as independent variables and cognitive impairment after severe stroke as the dependent variable, a single-factor binary logistic regression analysis was performed. The results showed that the number of CMB, the number of PVS, the number of cavities, WMH Fazekas score, and total CSVD score were significantly correlated with cognitive dysfunction (*P* < 0.05).

**Figure 10:**
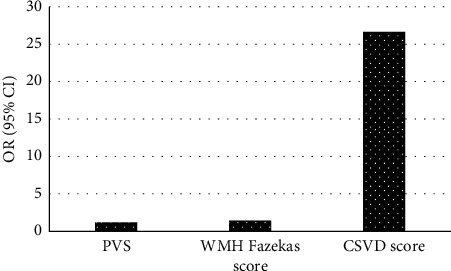
Multivariate binary logistic regression analysis.

Figure 10 illustrates the subsequent multivariate binary logistic regression analysis. With the number of CMB, the number of PVS, the number of cavities, the WMH Fazekas score, and the total CSVD score as independent variables and cognitive impairment after severe stroke as the dependent variable, the multivariate binary logistic regression analysis showed that the number of PVS, WMH Fazekas, and total CSVD score were independent factors of cognitive impairment (*P* < 0.05).

### 3.6. Correlation Analysis of MRI Feature Score and Cognitive Domain

**Figure 11:**
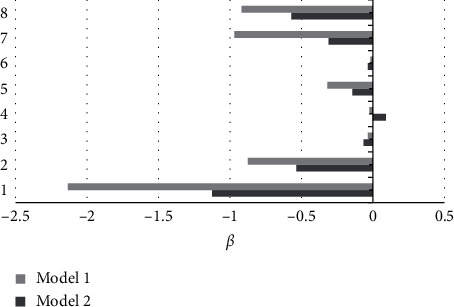
Linear regression model analysis. 1: MOCA score; 2: visual space and executive function; 3: naming; 4: abstract; 5: orientation; 6: speech; 7: attention; 8: memory; model 1: unadjusted risk factors; model 2: adjusted risk factors.

From Figure 11, the linear regression model analysis shows that when the risk factors were not adjusted, the MOCA score, visual space and executive function, orientation, attention, and memory were all significantly negatively correlated (*P* < 0.05). After adjustment of risk factors, only MOCA score, visual space and executive function, attention, and memory were significantly negatively correlated (*P* < 0.05).

## 4. Discussion

Stroke is a cerebrovascular disease with high morbidity, disability, and mortality. As revealed by research, the prevalence of cognitive impairment in patients with severe stroke was as high as 37% to 80%, and severe cases can be fatal [15]. MRI is widely used in the inspection and diagnosis of CSVD and has significant advantages. However, it will be affected by objective factors such as patient's breathing rate and doctor's operation in actual operation. The quality of the final MRI image is poor, so it is imperative to select a suitable image segmentation processing algorithm [16]. Therefore, a new image segmentation algorithm was proposed based on U-Net algorithm and conditional random field algorithm and was compared with FCN algorithm and U-Net algorithm. It was found that the ASSD (3.13 ± 1.35), HD (28.71 ± 9.05), Dice coefficient (0.78 ± 1.35), accuracy (0.74 ± 0.11), and sensitivity (0.85 ± 0.13) of this algorithm were superior to those of the other two algorithms. Such results suggested that the algorithm can automatically segment the stroke lesions, and it had the smallest segmentation error and the highest segmentation accuracy.

Then, the image segmentation algorithm based on U-Net and conditional random field was applied to the MRI diagnosis of 208 patients with severe stroke in our hospital to explore its impact on the mental state of patients with severe stroke. The Spearman rank correlation analysis revealed that the MOCA scores of 0 months (*r*_*s*_ = −0.675, *P*=0.000), 1 month (*r*_*s*_ = −0.715, *P*=0.000), and 3 months (*r*_*s*_ = −0.655, *P*=0.000) were negatively correlated with the total CSVD score, all with considerable significance (*P* < 0.05). After linear regression analysis, it was found that there was a significant negative correlation between the total CSVD score and the MOCA score, and the explanation had the highest proportion (0.702). However, the proportion of individual scores explaining cognitive impairment was generally low. Such result was the same as that of Del Brutto et al. [17] on the correlation between cognitive impairment and CSVD score. Single-factor binary logistic regression analysis of cognitive impairment group in severe stroke patients showed that the number of CMB, PVS, lacuna, WMH Fazekas score, and total CSVD scores all had a significant correlation (*P* < 0.05). Multivariate binary logistic regression analysis showed that the number of PVS, WMH Fazekas score, and total CSVD score were independent factors of cognitive impairment in severe brain, which was consistent with the results of the Sivakumar et al. [18], indicating that patients with severe stroke combined with the CSVD scores of the above items were relatively more likely to have cognitive impairment after stroke. After linear regression model analysis and correcting for risk factors, it was found that only the MOCA score, visual space and executive function, attention, and memory were significantly negatively correlated (*P* < 0.05), which was basically consistent with the research results of Liang et al. [19] on the correlation between total MRI cerebrovascular disease and cognitive impairment, indicating that it mainly affected attention, memory, and visual space and executive function.

## 5. Conclusion

In this research, a novel image segmentation algorithm was proposed based on the U-Net algorithm and the conditional random field algorithm and was compared with the FCN algorithm and the U-Net algorithm. Then, the image segmentation algorithm based on U-Net and conditional random field was applied to the MRI diagnosis of 208 patients with severe stroke in our hospital to explore the impact of cerebrovascular burden on the mental state of patients with severe stroke. It was found that the proposed algorithm can automatically segment stroke lesions with the smallest segmentation error and the highest segmentation accuracy. In addition, the number of PVS, WMH Fazekas score, and total CSVD score were independent factors of severe cognitive impairment after severe stroke, which mainly affected attention, memory, and visual space and executive function. The overall CSVD score explained cognitive decline after severe stroke in a significantly higher proportion than the CSVD alone. Therefore, clinicians should pay close attention to patients' CSVD when treating patients with severe stroke. Moreover, follow-up of cognitive function should be carried out, so as to realize early awareness and early intervention for patients with cognitive impairment as far as possible. However, there are still some problems to be solved in this study. There are too few training samples for the MRI image segmentation algorithm, and its performance and possible shortcomings need to be further discussed in the future. Moreover, there are few indicators to evaluate the characteristics of brain domains in stroke patients, which also requires more in-depth analysis. In conclusion, this study provides a scientific theoretical basis for clinical diagnosis and treatment and psychological nursing intervention of patients with severe stroke.

## Figures and Tables

**Table 1:** Brain MRI imaging features and CSVD scoring criteria [12].

Feature	Imaging features	Scoring criteria	Score
WMH	*T*1 showed isointensity or low intensity; *T*2 or *T*2 FLAIR showed hyperintensity, followed by Fazekas scale (0–6 points) scoring: paraventricular white matter hyperintensity (PVWMH) scoring and deep white matter hyperintensity (DWMH) scoring.	DWMH (Fazekas 2/3 points) and/or PVWMH (Fazekas 3 points)	1
CMB	There were round or oval foci in SWI or *T*2 ^*∗*^ GRE with signal loss (2∼5 mm < *D* < 10 mm) but not in other sequences.	≥1 CMB	1
PVS	The gap surrounded the blood vessel or run parallel to the blood vessel, in a line, round, or oval, *D* < 3 mm, *T*1 and FLAIR showed low signal, and *T*2 showed high signal.	PVS in the basal ganglia of grades 2–4	1
Lacuna	There was a lacuna under the round or oval cortex, 3 mm < *D* < 15 mm, and full of fluid, *T*I*W*I, *T*2*W*I, and FLAIR showed low signal.	≥1 lacuna	1
Total score			4

## Data Availability

No data were used to support this study.
